# The Evaluation of the Informational Content, Readability, and Quality of Online Information Related to Vitiligo in the Arabic Language

**DOI:** 10.7759/cureus.30497

**Published:** 2022-10-19

**Authors:** Logain Alghanemi, Saad A Sanad, Feras S Alzahrani, Esam A Hussien, Abdulaziz A Safi, Amal A Kokandi

**Affiliations:** 1 Dermatology, King Abdulaziz University Hospital, Jeddah, SAU; 2 Medicine, King Abdulaziz University Hospital, Jeddah, SAU; 3 Dermatology, Faculty of Medicine, King Abdulaziz University, Jeddah, SAU

**Keywords:** arabic, quality, readability, patient information, vitiligo

## Abstract

Background

Vitiligo is a common skin condition worldwide. It is an autoimmune disorder characterized by losing functional melanocytes, leading to chronic patchy depigmentation. People use the internet to seek health information, which is becoming one of the most commonly utilized sources. In this study, we aim to evaluate online information seen by patients about vitiligo by assessing the quality, content, and readability of widely used medical websites.

Methodology

All searches were conducted on February 16, 2022. The most popular search engines, Google, Yahoo, and Bing, were used to find websites, using vitiligo written in Arabic as a search term. An online readability calculator tool was used for the readability assessment of all websites. Two board-certified dermatology consultants (AK and LA) formulated a scoring sheet containing 19 questions based on commonly asked questions by patients in the dermatology clinics; 10 out of the 19 questions were designed to cover general information about vitiligo. In contrast, the other nine questions were designed to accommodate the management aspect of vitiligo. For the accountability assessment of each website, Journal of American Medical Association (JAMA) benchmarks were used. Statistical analysis has been performed using Statistical Package for the Social Sciences (SPSS) version 25 (IBM SPSS Statistics, Armonk, NY, USA).

Main measures

The following measures were used: a 19-question sheet, JAMA benchmarks, the Coleman-Liau index, and the Automated Readability Index (ARI).

Results

In this study, we analyzed 21 websites. The interobserver reproducibility was 0.946 between AK and LA (P < 0.001). For all websites, the mean (standard deviation (SD)) score of the questionnaire was 11.71 (3.45) (95% confidence interval (CI): 10.14-13.29) out of 19 possible points. Regarding all four JAMA benchmarks, no website achieved all benchmarks. Three of 21 websites (14.29%) completed three out of four JAMA benchmarks. No correlation was found between the content quality of the websites and JAMA benchmarks (r = 0.270, P = 0.237).

Conclusion

Online information about vitiligo in Arabic varies depending on the source, but overall, it is of low quality and written beyond the level of the general population. The “top 10 websites” outlined in our article may be used as a suggested reading list for vitiligo patients.

## Introduction

Vitiligo is a common skin condition affecting 0.5%-2% of people worldwide [1]. It is an autoimmune disorder characterized by losing functional melanocytes, leading to chronic patchy depigmentation [1-3]. A triggering factor leads to the initiation of an autoimmune response in genetically predisposed individuals that leads to the destruction of melanocytes. Both genders are equally affected, and it often begins between 10 and 30 years of age [2]. Vitiligo is often overlooked as a cosmetic issue. Its consequences may be emotionally distressing and significantly negatively impact self-esteem and social interaction [1,3]. Vitiligo presents significant dermatological care difficulties. Many methods are used to control vitiligo, such as topical and systemic immunomodulators, topical and systemic antioxidants, or phototherapy [2].

People use the internet to seek health information, which is becoming one of the most commonly utilized sources [4]. Almost 80% of all adult internet users in the United States of America (USA) have sought online information on various health conditions [5]. Of health seekers, 70% say the information they have found online has influenced their actions about how to treat themselves [6]. Nevertheless, until this point, there are no clear standards to assess the quality and accuracy of the data viewed. As a result, online information may be deficient, misleading, or leaning toward a commercial agenda. Therefore, the utility of these resources varies substantially depending on the source [7,8].

Many aspects of preserving health, and reaching for healthcare and health information, count upon comprehension of written knowledge. The term “health literacy” means “the degree to which individuals can obtain, process, and understand basic health information and services needed to make appropriate health decisions.” As defined by the National Assessment of Adult Literacy, the average adult person can read and understand at the eighth-grade level [8]. As a result, the United States (US) Department of Health and Human Services endorses that patient-oriented information must be written at a sixth-grade level or below. Nevertheless, health information presented on the internet may surpass the suggested reading level [9,10]. Many studies indicate that low health literacy is associated with miscommunication between patients and physicians, resulting in unfavorable health-related outcomes, such as an increased rate of in-hospital admissions, more visits to the emergency room for primary care reasons, increased mortality, and decreased screening for diseases [11]. As has been noted, it is of high value to study if the health information presented on the internet is correct and written at a suitable reading level. In this study, we aim to evaluate freely accessible online information about vitiligo by assessing the quality, content, and readability of commonly used medical websites using famous search engines.

## Materials and methods

**Figure 1:**
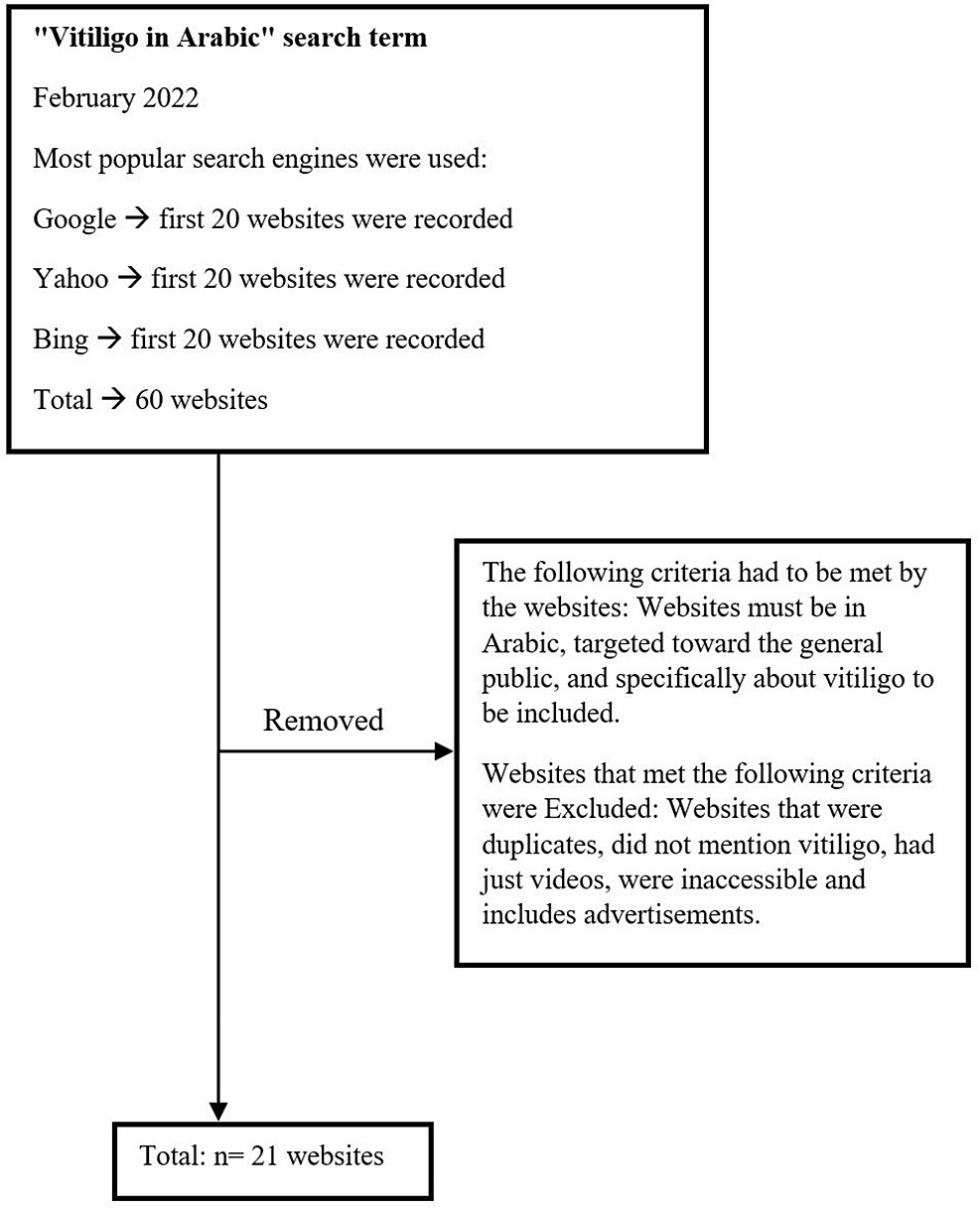
Flowchart for website selection

Ethical approval was not required for this study. All searches were conducted on February 16, 2022. Popular search engines, Google, Yahoo, and Bing, were used to find websites, using vitiligo written in Arabic as a search term. Cookies were deactivated, location information was erased, and users’ accounts were signed out to avoid unintentional search bias. The first 20 websites from each search engine were recorded. Websites must be in Arabic, targeted toward the general public, and explicitly covering vitiligo to be included. We excluded duplicate websites from other engines, inaccessible websites, and websites that contained only videos or advertisements (Figure 1). A total of 21 websites were collected for final analysis (Table 1).

**Table 1:** Lists of websites included for final analysis

Website	Link
Mayo Clinic	https://www.mayoclinic.org/ar/diseases-conditions/vitiligo/symptoms-causes/syc-20355912
WebTeb	https://www.webteb.com/dermatology/diseases/%D8%A7%D9%84%D8%A8%D9%87%D8%A7%D9%82
MOH	https://www.moh.gov.sa/HealthAwareness/EducationalContent/Diseases/Dermatology/Pages/011.aspx
Wikipedia	https://ar.wikipedia.org/wiki/%D8%A8%D9%87%D8%A7%D9%82
Jordan Finland Modern Hospital	https://jfmhospital.com/%D9%85%D8%B1%D8%B6_%D8%A7%D9%84%D8%A8%D9%87%D8%A7%D9%82_%D9%88%D8%B9%D9%84%D8%A7%D8%AC%D9%87/
Al-Tibbi	https://altibbi.com/%D9%85%D8%B5%D8%B7%D9%84%D8%AD%D8%A7%D8%AA-%D8%B7%D8%A8%D9%8A%D8%A9/%D8%A7%D9%84%D8%A7%D9%85%D8%B1%D8%A7%D8%B6-%D8%A7%D9%84%D8%AC%D9%84%D8%AF%D9%8A%D8%A9/%D8%A7%D9%84%D8%A8%D9%87%D8%A7%D9%82
Quartz Clinique	https://www.quartzclinique.com/ar/%D8%B9%D9%84%D8%A7%D8%AC-%D9%85%D8%B1%D8%B6-%D8%A7%D9%84%D8%A8%D9%87%D8%A7%D9%82-%D8%A7%D8%B3%D8%A8%D8%A7%D8%A8-%D8%A7%D9%84%D8%A8%D9%87%D8%A7%D9%82-%D9%88%D8%B9%D9%84%D8%A7%D8%AC%D9%87/
MSD Manuals	https://www.msdmanuals.com/ar/home/%D8%A7%D9%84%D8%A7%D8%B6%D8%B7%D8%B1%D8%A7%D8%A8%D8%A7%D8%AA-%D8%A7%D9%84%D8%AC%D9%84%D8%AF%D9%8A%D9%91%D9%8E%D8%A9/%D8%A7%D8%B6%D8%B7%D8%B1%D8%A7%D8%A8%D9%8E%D8%A7%D8%AA%D9%8F-%D8%B5%D9%90%D8%A8%D8%A7%D8%BA-%D8%A7%D9%84%D8%AC%D9%84%D8%AF%D9%90/%D8%A7%D9%84%D8%A8%D9%8F%D9%87%D8%A7%D9%82
Youm7	https://www.youm7.com/story/2020/3/5/%D9%85%D8%B1%D8%B6-%D8%A7%D9%84%D8%A8%D9%87%D8%A7%D9%82-%D9%88%D9%83%D9%84-%D9%85%D8%A7-%D8%AA%D8%B1%D9%8A%D8%AF-%D9%85%D8%B9%D8%B1%D9%81%D8%AA%D9%87-%D8%B9%D9%86-%D8%A3%D8%B9%D8%B1%D8%A7%D8%B6%D9%87-%D9%88%D8%B9%D9%84%D8%A7%D8%AC%D9%87/4657189
Youm7	https://www.youm7.com/story/2020/11/1/%D9%81%D9%89-%D8%A8%D9%8A%D8%AA%D9%83-%D9%85%D8%B1%D9%8A%D8%B6-%D8%A8%D9%87%D8%A7%D9%82-%D8%A7%D8%B9%D8%B1%D9%81-%D9%83%D9%84-%D8%AD%D8%A7%D8%AC%D8%A9-%D8%B9%D9%86-%D8%A7%D9%84%D9%85%D8%B1%D8%B6-%D9%88%D8%A5%D8%B2%D8%A7%D9%89/5047113
Mawdoo3	https://mawdoo3.com/%D8%A3%D8%B3%D8%A8%D8%A7%D8%A8_%D9%85%D8%B1%D8%B6_%D8%A7%D9%84%D8%A8%D9%87%D8%A7%D9%82
Enab Baladi	https://www.enabbaladi.net/archives/63710
Gate.ahram	https://gate.ahram.org.eg/News/2807980.aspx
Primo Medico	https://www.primomedico.com/ar/eilaj/lbhq/
BBC	https://www.bbc.com/arabic/science-and-tech-50463174
Al-Muwatin	https://www.almowaten.net/2021/08/%D8%A7%D9%84%D8%B3%D8%B9%D9%88%D8%AF%D9%8A%D8%A9-%D9%85%D9%86-%D8%A3%D9%81%D8%B6%D9%84-%D8%AF%D9%88%D9%84-%D8%A7%D9%84%D8%B9%D8%A7%D9%84%D9%85-%D9%81%D9%8A-%D8%B9%D9%84%D8%A7%D8%AC-%D8%A7%D9%84%D8%A8/
Docspert Health	https://www.docspert.com/blog/%d8%a7%d9%84%d8%a8%d9%87%d8%a7%d9%82-%d8%a7%d9%84%d8%ae%d9%81%d9%8a%d9%81/
Tajmeeli	https://tajmeeli.com/%D8%B9%D9%84%D8%A7%D8%AC-%D8%A7%D9%84%D8%A8%D9%87%D8%A7%D9%82/
Sawah Media	https://sawahmedia.com/articles/%D9%85%D8%A7-%D9%87%D9%8A-%D8%A7%D8%B9%D8%B1%D8%A7%D8%B6-%D9%85%D8%B1%D8%B6-%D8%A7%D9%84%D8%A8%D9%87%D8%A7%D9%82-%D9%88%D8%A7%D8%B3%D8%A8%D8%A7%D8%A8%D9%87%D8%9F/
Dar Al-Tib	https://www.daralteb.net/vitiligo/
Ts3a	https://www.ts3a.com/%D8%B9%D9%84%D8%A7%D8%AC-%D9%85%D8%B1%D8%B6-%D8%A7%D9%84%D8%A8%D9%87%D8%A7%D9%82/

Readability analysis

Each website text was formatted in Microsoft Word (Microsoft Corp., Redmond, WA, USA). We manually excluded figures, captions, hyperlinks, advertisements, and locations before starting to calculate readability. For the assessment of the readability of all websites, an online readability calculator tool was used [12] that was also used previously in the literature [13,14]. The calculator tool was used primarily for English text assessment. Nevertheless, it can also be implicated in assessing other languages. This website uses popular text-analyzing tools: the Coleman-Liau index (this index calculates the readability grade using the average number of characters per word and the average number of sentences per 100 words) and the Automated Readability Index (ARI) (this index compares the number of letters in a word to the number of words in a sentence). Other tools were not used as they are inapplicable for assessing Arabic-written texts; unlike English words, Arabic words are comprised of letters linked to each other and do not contain syllables that are included in the formulas of other readability indices. Both the Coleman-Liau index and ARI report a US reading grade level.

Content analysis

Two board-certified dermatology consultants (AK and LA) formulated a scoring sheet containing 19 questions based on commonly asked questions by patients in the dermatology clinics. Ten out of the 19 questions were designed to cover general information about vitiligo, while the other nine questions were designed to accommodate the management aspect of vitiligo. Those questions were designed to cover commonly conveyed information during the patient’s visit to the clinic. They were used to evaluate how complete and accurate the content of each website is. To assess the content of each website, a score of 0 and 1 was given for each question; 0 indicates the absence of information covered by a particular question, and 1 indicates the presence of information covered by a particular question. Each observer independently assessed each question, and inter-rater reliability was measured to confirm agreement. The mean (standard deviation (SD)) score among the two observers was used to compare the quality of each site.

For the accountability assessment of each website, Journal of American Medical Association (JAMA) benchmarks were used. This tool assesses the presence of four major components: authorship, attributions, disclosure, and currency. To fulfill the authorship requirements outlined by the JAMA, a website must include authors and contributors, their affiliations, and relevant credentials. All attributions, or references, should be listed, and all applicable copyright information should be noted. Website “ownership” should be prominently and fully disclosed, as should any sponsorship, advertisements, underwriting, or commercial funding. Dates that content was posted and updated should be stated.

Statistical analysis

Statistical analysis has been performed using Statistical Package for the Social Sciences (SPSS) version 25 (IBM SPSS Statistics, Armonk, NY, USA). Descriptive statistics were calculated from all data, using the median, mean, range, interquartile range (IQR), and confidence interval (CI), when applicable, for reporting. A correlation study was then performed using Pearson’s correlation coefficient between the content analysis score, readability score, and JAMA benchmarks. The intraclass correlation coefficient was used to measure inter‐rater reliability (absolute agreement). P < 0.05 was considered statistically significant.

## Results

Website selection and content analysis

**Figure 2:**
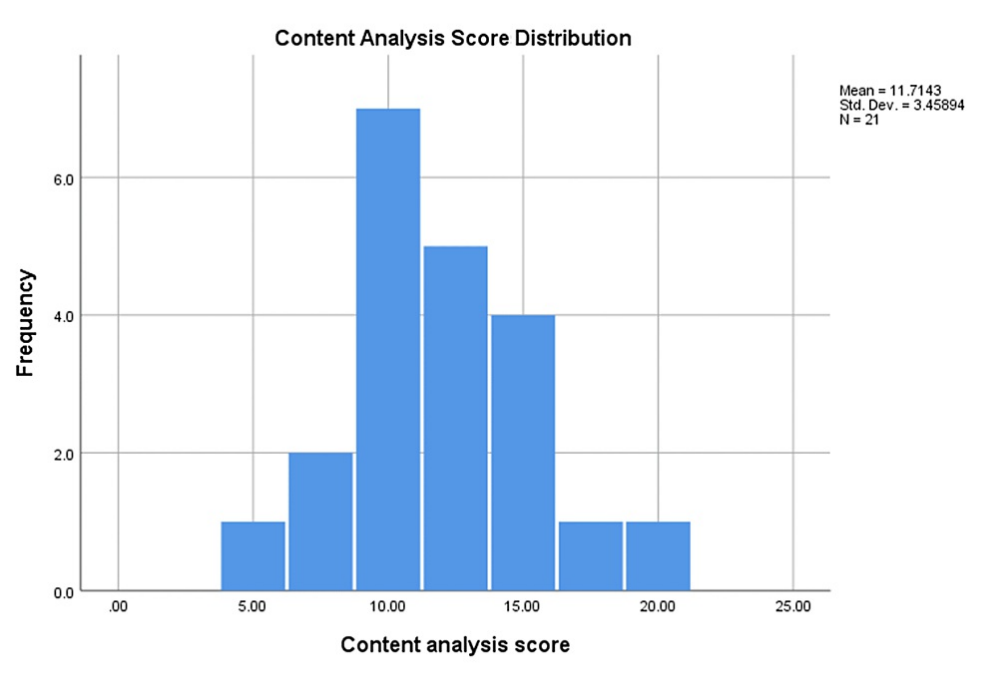
Website content analysis score

In this study, we analyzed 21 websites. The interobserver reproducibility was 0.946 between AK and LA (P < 0.001). For all websites, the mean (SD) score of the questionnaire was 11.71 (3.45) (95% CI: 10.14-13.29) out of 19 possible points. The top-scoring website was Mawdoo3, with a mean (SD) of 18.5, representing 97.37% of the total possible points. Gate.ahram was the website with the lowest score, with 5 points, representing 26.32% of the total possible points. Figure 2 illustrates the distribution of website content scores. Table 2 shows the scores of each question for the top 10 websites.

**Table 2:** Content analysis scores for the top 10 websites

	Mawdoo3	Tajmeeli	Mayo Clinic	WebTeb	MOH	Wikipedia	Al-Tibbi	Quartz Clinique	MSD Manuals	Enab Baladi
What is vitiligo?	1.0	1.0	1.0	1.0	1.0	1.0	1.0	1.0	1.0	1.0
What are the symptoms of vitiligo?	1.0	1.0	1.0	1.0	1.0	1.0	1.0	1.0	1.0	1.0
What is the age of onset?	1.0	1.0	1.0	0.5	0.5	1.0	1.0	1.0	0.0	1.0
What are the associated diseases?	1.0	1.0	0.5	1.0	1.0	1.0	1.0	1.0	1.0	1.0
What are the causes of vitiligo?	1.0	1.0	1.0	1.0	1.0	1.0	1.0	1.0	1.0	1.0
Can vitiligo be prevented?	1.0	0.0	0.5	1.0	1.0	0.0	0.5	0.0	0.0	0.5
Is vitiligo contagious?	1.0	1.0	1.0	0.0	1.0	0.0	1.0	1.0	0.0	0.0
How is vitiligo diagnosed?	1.0	1.0	1.0	1.0	1.0	0.0	1.0	0.5	1.0	0.0
What is the difference between vitiligo and leprosy?	0.5	0.5	0.0	0.0	0.0	0.0	0.0	0.0	0.0	0.0
What is the course of the disease?	1.0	1.0	1.0	1.0	1.0	1.0	1.0	0.5	0.0	1.0
What are the different treatment modalities?	1.0	1.0	1.0	1.0	1.0	1.0	1.0	1.0	1.0	1.0
What are the types most likely unresponsive to treatment?	1.0	0.0	0.0	0.0	1.0	0.0	0.0	0.0	0.0	0.0
What are the topicals used in vitiligo?	1.0	1.0	1.0	1.0	1.0	1.0	1.0	1.0	1.0	0.5
What are the different procedures that can be used?	1.0	1.0	1.0	1.0	1.0	1.0	1.0	1.0	1.0	0.5
What is phototherapy?	1.0	1.0	1.0	1.0	1.0	1.0	0.5	1.0	1.0	1.0
What is depigmentation therapy?	1.0	1.0	1.0	1.0	0.0	1.0	0.0	0.0	1.0	1.0
When can depigmentation be used?	1.0	1.0	1.0	1.0	0.0	1.0	0.0	0.0	1.0	1.0
What is the prognosis of the disease?	1.0	1.0	1.0	1.0	1.0	1.0	0.5	0.5	0.5	0.0
Does the source show picture(s) of vitiligo?	1.0	1.0	1.0	1.0	0.0	1.0	1.0	1.0	1.0	1.0
Total (number (%))	18.5 (97.37)	16.5 (86.84)	16 (84.21)	15.5 (81.58)	14.5 (76.32)	14 (73.69)	13.5 (71.05)	12.5 (65.79)	12.5 (65.79)	12.5 (65.79)

Of the 19 quality questions, the questions with the highest score were as follows: question 1 (What is vitiligo?), question 2 (What are the symptoms of vitiligo?), and question 11 (What are the different treatment modalities?). The lowest-scoring question was question 9 (What is the difference between vitiligo and leprosy?). The average score for each question is summarized in Table 3.

**Table 3:** Average score for each question

Question	Number of websites (N = 21) and percentage (%)
What is vitiligo?	21 (100%)
What are the symptoms of vitiligo?	21 (100%)
What is the age of onset?	13 (62%)
What are the associated diseases?	11 (52%)
What are the causes of vitiligo?	21 (100%)
Can vitiligo be prevented?	7 (33%)
Is vitiligo contagious?	12 (57%)
How is vitiligo diagnosed?	12 (57%)
What is the difference between vitiligo and leprosy?	1 (4%)
What is the course of the disease?	13 (61%)
What are the different treatment modalities?	21 (100%)
What are the types most likely unresponsive to treatment?	3 (14%)
What are the topicals used in vitiligo?	16 (76%)
What are the different procedures that can be used?	13 (61%)
What is phototherapy?	17 (80%)
What is depigmentation therapy?	9 (42%)
When can depigmentation be used?	8 (38%)
What is the prognosis of the disease?	10 (47%)
Does the source show picture(s) of vitiligo?	20 (95%)

Regarding all four JAMA benchmarks, no website achieved all JAMA benchmarks. Three of the 21 websites (14.29%) completed three of the four JAMA benchmarks (Table 4). Authorship and currency were the most frequently viewed benchmarks. No correlation was found between the websites’ content quality and JAMA benchmarks (r = 0.270, P = 0.237).

**Table 4:** JAMA benchmark score for websites

JAMA benchmark	Number (%) (N = 21)
Number of benchmarks	
4.0	0
3.0	3 (14.3)
2.0	9 (42.86)
1.0	6 (28.57)
0.0	3 (14.3)
Attribution	10 (47.62)
Authorship	7 (33.3)
Currency	17 (80.95)
Disclosure	5 (23.81)

Readability

The mean (SD) grade level for all websites was 13.05 (11.92) (95% CI: 10.63-15.47). No correlation was found between the Coleman-Liau index and the ARI index (r = 0.461, P = 0.035). Mayo Clinic had the lowest US grade of 7.92, and Ts3a had the highest US grade level of 28.76. None of the websites achieved the recommended sixth-grade level or below. No significant correlation was found between website content quality and the mean reading grade (r = 0.177, P = 0.443). Table 5 shows the content analysis scores of the top 10 websites and their readability grade.

**Table 5:** Top 10 website content analysis scores and readability grades

Website	Content analysis score (number (%))	Readability (grade level)
Mawdoo3	18.5 (97.37)	23.83
Tajmeeli	16.5 (86.84)	12.85
Mayo Clinic	16 (84.21)	7.92
WebTeb	15.5 (81.58)	11.01
MOH	14.5 (76.32)	8
Wikipedia	14 (73.69)	11.38
Al-Tibbi	13.5 (71.05)	13.82
Quartz Clinique	12.5 (65.79)	9.01
MSD Manuals	12.5 (65.79)	14.19
Enab Baladi	12.5 (65.79)	17.35

## Discussion

Patients’ use of online resources to gain knowledge regarding their medical conditions and treatment choices has substantially increased [15-18]. Online resources provide cheap and easily accessible health information that helps patients make decisions [19,20]. Of medical oncology physicians, 50% reported a negative impact of online medical material on their patients, as it could be related to a large amount of content and information inaccuracy [21,22]. On another note, a British study has found that almost half of patients who learn information from online sources do not share it with their care providers [23]. Online resources should be readable, reliable, and accurate to help patients make decisions regarding their medical conditions.

The assessment of the content quality of 21 websites showed that the average score was 11.71 out of 19 possible points (61.85%), indicating poor quality of the information provided online as a reference for patients. According to Eysenbach and Köhler, many readers determine the credibility of given information after viewing only one website online [24]. Accordingly, given the narrow scope of free Arabic online information about vitiligo, the poor average score of websites in our study (61.85%) may lead patients toward incorrect information regarding vitiligo symptoms, diagnostic modalities, or treatment options, and it could cause adverse health and related psychosocial outcomes. No website achieved all four JAMA benchmarks; three websites (Mayo Clinic, Wikipedia, and Mawdoo3) achieved three out of the four benchmarks. The average displayed benchmark for all websites was 2, indicating less-than-ideal content accountability. The disclosure was the least displayed JAMA benchmark, as only five out of 21 websites achieved it. Disclosure is a fundamental entity; research by McD Taylor et al. showed the importance of referencing as it helps in papers’ validations and text readability and helps orient the reader toward correct information [25]. Accordingly, we think the lack of disclosure on most websites decreases the paper’s accountability and readability and may mislead readers toward inaccurate resources. No correlation was found between content quality and JAMA benchmarks, as it could mean websites with wrong information may seem of better quality and taken as more accurate.

The average reading grade across the 21 websites was 13.05 US reading level grade, which is higher than the recommended sixth grade. These data suggest that information regarding vitiligo on the internet is addressed toward higher-educated individuals and is unlikely to be understood by the general public. Mayo Clinic was the least complex website, with a readability score correlated to eighth-grade reading level. Despite ranking third in content analysis, no correlation was found between grade reading level and average content analysis score. Readability testing is significant because it determines the level of written content, which helps medical writers discover places that may need to be adjusted to improve text comprehension.

This study has some inherent limitations. The readability test does not cover background information, graphics, and the layout of the page. Also, the website used for readability calculation was originally designed to calculate the readability of English texts, not Arabic.

The content analysis questionnaire also has limitations. The score is dependent on observer subjectivity, which may lead to bias. To minimize this, two different observers assessed the websites with high interobserver reliability. Furthermore, the content analysis questionnaire does not consider how information is displayed or how simple it is to search and discover information on a particular website. Another limitation that needs to be addressed is that patient comprehension of vitiligo as a result of reading the content supplied was not assessed and compared to our evaluation of each website. Additionally, it only covers the availability of the predetermined data and not qualifying the information. Lastly, this study did not assess the presence of incorrect additional information. More studies are needed to investigate how patients look for information regarding medical conditions in Arabic and what knowledge they get after reading each website.

## Conclusions

The presented study found that online information about vitiligo in Arabic varies depending on the source, but overall, it lacks quality. Moreover, the information available online in the Arabic language is written beyond the level of the general population, which may lead to understanding difficulties. Most of the websites reviewed did not have sufficient information to aid patients in making medical decisions. Despite these limitations, there is a wide range of online resources on vitiligo. The “top 10 websites” outlined above may be used as a suggested reading list for vitiligo patients.
